# An immune response gene expression module identifies a good prognosis subtype in estrogen receptor negative breast cancer

**DOI:** 10.1186/gb-2007-8-8-r157

**Published:** 2007-08-02

**Authors:** Andrew E Teschendorff, Ahmad Miremadi, Sarah E Pinder, Ian O Ellis, Carlos Caldas

**Affiliations:** 1 Breast Cancer Functional Genomics Laboratory, Cancer Research UK Cambridge Research Institute and Department of Oncology, University of Cambridge, Robinson Way, Cambridge CB2 0RE, UK; 2 Cambridge Breast Unit, Addenbrookes Hospital, Cambridge University Hospitals NHS Foundation Trust, Hills Road, Cambridge, CB2 0QQ, UK; 3 Histopathology, Nottingham City Hospital NHS Trust and Department of Pathology, University of Nottingham, Nottingham NG5 1PB, UK

## Abstract

**Background:**

Estrogen receptor (ER)-negative breast cancer specimens are predominantly of high grade, have frequent p53 mutations, and are broadly divided into HER2-positive and basal subtypes. Although ER-negative disease has overall worse prognosis than does ER-positive breast cancer, not all ER-negative breast cancer patients have poor clinical outcome. Reliable identification of ER-negative tumors that have a good prognosis is not yet possible.

**Results:**

We apply a recently proposed feature selection method in an integrative analysis of three major microarray expression datasets to identify molecular subclasses and prognostic markers in ER-negative breast cancer. We find a subclass of basal tumors, characterized by over-expression of immune response genes, which has a better prognosis than the rest of ER-negative breast cancers. Moreover, we show that, in contrast to ER-positive tumours, the majority of prognostic markers in ER-negative breast cancer are over-expressed in the good prognosis group and are associated with activation of complement and immune response pathways. Specifically, we identify an immune response related seven-gene module and show that downregulation of this module confers greater risk for distant metastasis (hazard ratio 2.02, 95% confidence interval 1.2-3.4; *P *= 0.009), independent of lymph node status and lymphocytic infiltration. Furthermore, we validate the immune response module using two additional independent datasets.

**Conclusion:**

We show that ER-negative basal breast cancer is a heterogeneous disease with at least four main subtypes. Furthermore, we show that the heterogeneity in clinical outcome of ER-negative breast cancer is related to the variability in expression levels of complement and immune response pathway genes, independent of lymphocytic infiltration.

## Abstract 2

A feature selection method was used in an analysis of three major microarray expression datasets to identify molecular subclasses and prognostic markers in estrogen receptor-negative breast cancer, showing that it is a heterogeneous disease with at least four main subtypes.

## Background

It is widely recognized that estrogen receptor (ER)-positive (ER^+^) and ER-negative (ER^-^) breast cancers are two different disease entities. Generally, ER^- ^tumours tend to be of high grade, are more frequently p53 mutated, and have worse prognosis compared with ER^+ ^disease. Moreover, while ER^+ ^disease can be treated with hormone therapy, the only targeted therapy available for ER^- ^patients is a monoclonal antibody that binds to the *ERBB2 *receptor and that is effective only for those ER^- ^tumours with *HER2*/*ERBB2 *over-expression.

In spite of these clinical advances, ER^+ ^and ER^- ^breast cancers remain heterogeneous diseases, and little is known regarding why patients with the same histopathologic characteristics may have widely different clinical outcomes [1]. This is particularly true for the basal subtype of ER^- ^breast cancer, which is commonly defined by over-expression of cytokeratin markers (CK 5/6 and CK 14) and which is often also HER2 negative (HER2^-^) [2]. Most recent efforts to obtain a molecular understanding of the observed heterogeneity have focused on ER^+ ^breast cancer, where gene expression signatures that are either prognostic or predictive of response to hormone therapy have been derived [3-10]. In contrast, few studies have thus far attempted to derive a prognostic signature within ER^- ^breast cancer. Although cytokeratin markers have been shown to correlate with poor prognosis in breast cancer [11-14], an attempt to correlate basal markers with survival within ER^- ^disease has shown that these markers were not predictive of outcome [15]. Based on our work presented here, it appears that the prognostic 'signal' in ER^- ^breast cancer is much weaker than that in ER^+ ^disease. This precludes the use of traditional supervised approaches, which assume a sufficiently low false discovery rate (FDR) for deriving gene expression based classifiers. A similar observation was reported by others [16].

Recently, we proposed a novel feature selection method (Profile Analysis using Clustering and Kurtosis [PACK]) [17], that selects genes using a pattern recognition method and that may significantly reduce the FDR. Using PACK in an integrated cohort of 186 ER^- ^samples and 1,200 genes, we were able to identify distinct molecular subtypes, including a good prognosis subclass characterized by over-expression of immune response genes. However, these results were not validated in external cohorts.

The purpose of this work is to extend our preliminary findings [17] by applying PACK to the same three breast cancer datasets [5,9,18], but now using a much larger set of common genes (we rescued a significantly larger number of genes from our study [9] by imputing missing data, which led to a much larger number of overlapping genes, approximately 5,000, with the other two arrays.), and to further validate our findings using two external independent cohorts [7,19]. More generally, our goal is to elucidate the molecular taxonomy of ER^- ^breast cancer and, if possible, to find different prognostic subclasses and the corresponding prognostic markers.

## Results

### The FDR is higher in ER^- ^breast cancer

**Figure 1:**
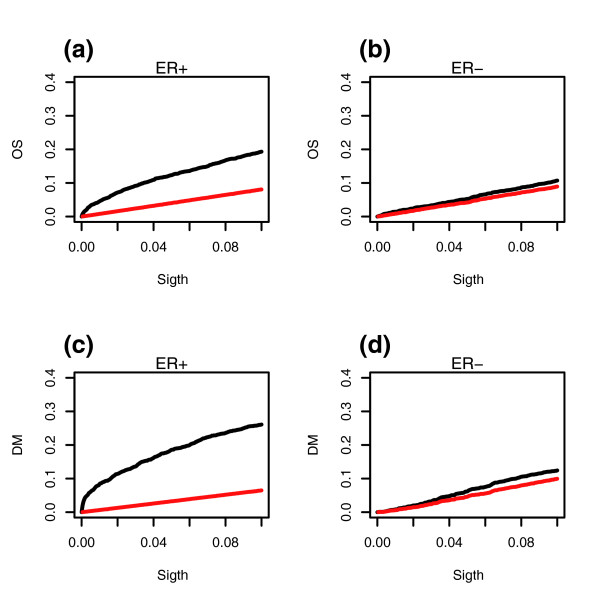
FDR comparison in ER^- ^and ER^+ ^breast cancer. For various significance thresholds (sigth), we plot the fraction of observed genes with *P *values less than the significance threshold (black) as well as the corresponding fraction of false positives, as estimated using a q value analysis (red). **(a) **Overall survival for ER^+ ^breast cancer. **(b) **Overall survival for ER^- ^breast cancer. **(c) **Time to distant metastasis for ER^+ ^breast cancer. **(d) **Time to distant metastasis for ER^- ^breast cancer. *P *values were obtained from the log-rank test using Cox regression models. ER, estrogen receptor; FDR, false discovery rate.

To understand why in ER^- ^breast cancer it has not been possible to derive a validated prognostic signature using conventional approaches, we compared the FDR for ER^+ ^and ER^- ^disease. We used as cohorts integrated datasets obtained by merging together three of the largest profiled breast cancer cohorts [5,9,18] using the z-score transformation, a procedure that we validated previously [17]. Briefly, the z-score transformation shifts the mean of each gene expression vector in each cohort to zero, while scaling its variance to unity. The transformed gene expression vectors are then merged across cohorts. This merging step resulted in integrated expression matrices of 186 ER^- ^and 527 ER^+ ^tumors profiled over a common set of 5,007 genes. To enable the comparison, we selected at random 186 ER^+ ^tumors from the 527 available. We then used the univariate Cox proportional hazards model with time to distant metastasis (TTDM) and overall survival as end-points to obtain *P *values of significance for all the genes. Next, we estimated, for each choice of significance threshold, the number of false positives using the q-value approximation [20]. The numbers of significant genes and false positives as a function of the significance threshold were then plotted for ER^+ ^and ER^- ^breast cancer and for the two different end-points used (Figure 1). We verified that the curves for ER^+ ^breast cancer were robust to random selections of the 186 ER^+ ^tumors. This showed that the FDR is much higher in ER^- ^tumours and motivated us to develop a different feature selection approach based on a pattern recognition algorithm (PACK) [17].

### Finding ER^- ^subclasses using PACK

If the aim is to identify subclasses within a tumor type, then it is natural that unsupervised methods be applied to sample sets that are composed entirely of this tumor type. In fact, given the hierarchical ER^+^/ER^- ^subdivision for breast cancer, it is questionable whether ER^- ^subgroups can be correctly defined based on clusters that were derived by using both ER^+ ^and ER^- ^samples, as was done in other studies (for example [21-23]). To see this, assume that all tumor types and genes are used in the clustering algorithm. It is very likely that the clusters within ER^- ^tumors reflect not only the interesting variability of genes within ER^- ^tumors but also the variability of genes that are important for classification of ER^+ ^tumors. The variability of these genes in ER^- ^tumors may represent undesired noise, which, if not removed, can affect the inferred clusters. Thus, in order to identify relevant subtypes of ER^- ^tumors more robustly, we decided to use ER^- ^tumors only. Moreover, in view of the relatively high FDR in ER^- ^disease, we decided to apply the PACK methodology, which has already been shown to provide a more reliable identification of molecular classifiers [17]. Because PACK requires large sample sizes (for small sample sizes, kurtosis estimates can have large variance and clustering algorithms that aim to predict the optimal number of clusters have a high false negative rate), we applied it to the integrated data matrix derived previously. Two additional independent ER^- ^cohorts [7,19] were left out to serve as validation studies. The five microarray datasets used are summarized in Table 1 by platform type, number of ER^- ^samples, and number of poor outcome events.

**Table 1:** Breast cancer datasets used

Ref.	Cohort name	Oligo microarray platform	ER^-^		ER^+^	
			
			*n*	Death/distant metastasis (*n*)	*n*	Death/distant metastasis (*n*)
[18]	NKI2	Agilent	69	34	226	45
[5]	EMC	Affymetrix	77	27	208	80
[9]	NCH	Agilent	40	14	93	21
[19]	UPP	Affymetrix	34	6	213	49
[7]	JRH-2	Affymetrix	24	6	72	17

ER, estrogen receptor.

**Figure 2:**
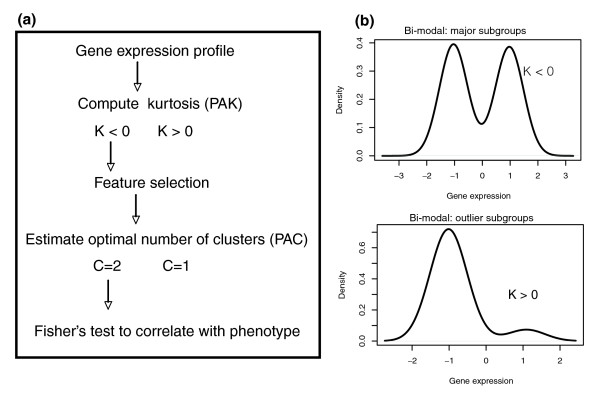
PACK flowchart. **(a) **A schematic diagram of PACK, as used in this study. For each gene expression profile an unbiased estimate of its kurtosis, K, is computed. Genes with negative kurtosis are selected because only these define large subgroups (of sizes >22% of the total sample size). Further unsupervised clustering may then be performed on this subset of negative kurtosis profiles to find novel tumor subclasses. Alternatively, to find robust prognostic markers, negative kurtosis profiles are filtered further based on whether there is evidence of bimodality (C = 2). This step requires a cluster inference algorithm and a model selection criterion to discard those profiles that are best described by a single gaussian (C = 1; by random chance gaussian profiles may have negative kurtosis). Correlation to phenotypes (here phenotypes) is done with Fisher's test to evaluate whether the distribution of the categorical phenotype across the two clusters is significantly different from random. **(b) **Density curves of typical bimodal negative and positive kurtosis gene expression profiles. X-axis shows gene expression on a log2 scale. PACK, Profile Analysis using Clustering and Kurtosis.

Briefly, we review the concepts that underpin PACK (see Materials and methods, below, and Figure 2). The hypothesis is that genes that play an important role as classifiers or biomarkers are more likely to have expression profiles that are mixtures of gaussian distributions. On the other hand, false positives, in spite of their spurious association with a phenotype, are less likely to be described by a mixture of distributions. Thus, selecting genes based on whether they have structure in their expression profiles is likely to pick out the relevant markers from those that are just false positives. Next, we propose to focus on those genes that define the largest subgroups. Although genes that define small subgroups are also of interest, it is natural to identify first those genes that define the largest subclasses. While such features can be found from the inferred cluster sizes, it turns out that such features are generally also characterized by a negative (or close to zero) kurtosis profile (see Materials and methods, below) [17]. As shown previously, negative kurtosis expression profiles are in effect a mixture of at least two (gaussian) distributions of approximately equal weights. Thus, by selecting those genes that have the most negative kurtosis expression profiles, we have identified the markers that define the largest subclasses within the sample set (Figure 2). It is clear that many of these features will be highly correlated because they define almost the same subclasses. It follows that further application of traditional clustering algorithms over these negative kurtosis profiles will enable reliable identification of the major subclasses within the sample set. We note that because we are interested in the most negative kurtosis profiles and because the clusters in the individual gene profiles are only needed to study the cluster distribution of phenotypes, the cluster inference step on the individual gene profiles (known as PAC) can be performed after computation of kurtosis (known as PAK) on the selected subset of negative kurtosis profiles (Figure 2).

### Distinct molecular subgroups of ER^- ^breast cancer

Applying PAK to the integrated ER- data matrix of 5,007 genes, we found 813 genes with a negative kurtosis profile (Additional data file 1). Interestingly, applying the same analysis to ER^+ ^breast cancer, we found only a much smaller number (193) of negative kurtosis profiles (Additional data file 2), despite there being roughly twice as many bimodal profiles in ER^+ ^breast cancer (about 4500 in ER^+ ^versus about 2,500 in ER^-^). We verified by explicit simulation that the significantly lower proportion of negative kurtosis profiles in ER^+ ^disease could not be explained by the larger sample size in ER^+ ^(527) compared with ER^- ^disease (186; data not shown).

**Figure 3:**
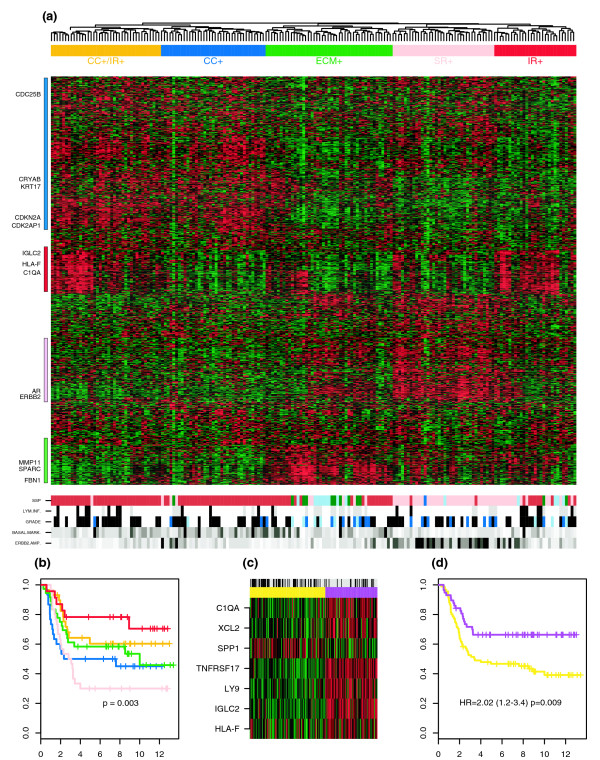
Molecular subclasses in ER^- ^breast cancer. **(a) **Complete linkage hierarchical clustering of 186 ER^- ^breast tumors over 813 genes with negative kurtosis profiles. Five sample clusters were identified and characterized in terms of the patterns of over-expression and under-expression of four gene clusters related to cell cycle (CC; blue), immune response (IR; red), extracellular matrix (ECM; green), and steroid hormone response (SR; pink) functions. Panels show the distribution of the SSP subtype [23], the lymphocytic infiltration score, histologic grade, basal marker [27], and *ERBB2*^+ ^amplifier subtype. Panel color codes: SSP (pink = HER2, brown = basal, dark green = normal, sky blue = luminal A, and blue = luminal B); LYM.INF (black = high, gray = low, and white = missing); GRADE (black = high, blue = intermediate, sky blue = low, and white = missing), BASAL.MARK. (black = high and white = low), ERBB2-AMP (black = high and white = low). The BASAL.MARK. profile represents an average over validated basal markers in [27], whereas the ERBB2-AMP profile was calculated as an average over three genes in the *ERBB2 *amplicon (*ERBB2*, *STARD3*, *GRB7*). **(b) **Kaplan-Meier curves for time to distant metastasis (years) and for the five subclasses identified in panel (a). **(c) **Partitioning around medoids clustering over the seven-gene prognostic immune response module. Panel color codes: purple = cluster over-expressing module, yellow = cluster under-expressing module, black = poor outcome samples, gray = good outcome samples, green = relative under-expression, and red = relative over-expression. **(d) **Kaplan-Meier curves for time to distant metastasis for the two groups identified in panel (c). Hazard ratio, 95% confidence interval, and log-rank test *P *values are shown. ER, estrogen receptor; SSP, single sample predictor.

Having identified the relevant features, we next clustered the ER^- ^tumors over these. Using hierarchical clustering with Pearson correlation metric and complete linkage, we found that samples clustered into five main groups, each characterized by the expression patterns of four gene clusters that were found to be strongly enriched for specific gene ontologies (Figure 3a and Additional data file 3). One group was characterized by over-expression of *ERBB2*, the steroid hormone receptor *AR*, and genes related to steroid estrogen response (such as *GATA3*, *TFF1*, and *DNALI1*). This subtype is therefore similar to the apocrine subclass, recently proposed [24,25], which is characterized by over-expression of *AR *and genes that are either direct targets of ER or responsive to estrogen [24,25]. Thus, we decided to call this ER^- ^subtype (over-expressing steroid response genes) 'SR^+^', although it is clear that it also defines the well known HER2^+ ^subtype.

The other four groups were characterized mainly by absent or lower expression of these steroid response genes. One of these four clusters was characterized by over-expression of genes related to cell cycle and cell proliferation pathways (CC^+^), and another cluster also had over-expression of immune response genes (CC^+^/IR^+^). For the remaining two clusters, one was characterized by over-expression of extracellular matrix genes (ECM^+^), and the other was characterized by over-expression of immune response genes (IR^+^) only.

### Relation to the intrinsic subtype classification

In order to relate the five identified molecular subtypes to the intrinsic breast cancer classification [21,26], we used the recently validated single sample predictor (SSP) [23] to classify the 186 ER^- ^samples into the various intrinsic subtypes (Figure 3a and Table 2). In addition, we studied the expression profiles of recently validated basal markers [27] and genes in the *ERBB2 *amplicon across the five identified clusters (Figure 3a and Additional data file 4). Based on these figures and Table 2, we could draw the following conclusions. First, the SR^+ ^cluster was highly correlated with the usual HER2^+ ^intrinsic subtype. Second, the CC^+^/IR^+ ^and CC^+ ^clusters defined distinct subtypes of basal tumors. Third, the ECM^+ ^cluster was mostly basal, but it contained a relatively high proportion of normal-like and luminal-A-like ER^- ^tumors; it also exhibited the most varied grade distribution, with most low-grade ER^- ^tumors falling into this class. Finally, the main constituents of the IR^+ ^cluster were basal and HER2^+^.

**Table 2:** Distribution of ER^- ^samples among clusters, prognostic groups and intrinsic subtypes

Cluster	*n*	Outcome		LI		ER^-^				
		Good	Poor	High	Low	Luminal B	Luminal A	Normal	Basal	HER2
CC^+^/IR^+^	39	25	14	7	2	0	0	0	38	1
CC^+^	37	18	17	3	5	0	0	1	33	3
ECM^+^	45	26	18	1	14	0	9	9	22	5
SR^+^	36	16	20	2	4	1	1	0	2	32
IR^+^	29	23	6	6	6	0	4	2	9	14

For the five clusters of Figure 2a, we give the number of samples per cluster (*n*) and their distributions over good/poor outcome, high/low lymphocytic infiltration (LI) score, and intrinsic subtypes as determined by the single sample predictor (SSP) classifier. Clusters were labeled by the main Gene Ontology of genes over-expressed in the group: CC^+ ^(cell cycle), CC^+^/IR^+ ^(cell cycle and immune response), ECM^+ ^(extracellular matrix), SR^+ ^(steroid hormone response), and IR^+ ^(immune response). ER, estrogen receptor.

It further follows from these observations that ER^- ^normal and luminal A samples were predominantly characterized by over-expression of ECM genes. ER^- ^basal tumors, on the other hand, exhibited a more complex pattern and appeared to divide into at least four subgroups (CC^+^/IR^+^, CC^+^, ECM^+^, and IR^+^).

To investigate further the relation of our five ER- subclasses with the intrinsic subtypes, we considered to which ER^- ^subclass ER^+ ^samples of known intrinsic subtype were most similar. To this end we first constructed, for each of the five ER^- ^subclasses, mean centroids over the 813 negative kurtosis genes (Additional data file 5). To validate the centroids, the same ER^- ^samples were assigned a subclass using a nearest centroid criterion (samples for which the largest Pearson correlation coefficient was <0.25 were considered unclassified; see Materials and methods, below), which showed that 156 (84%) were classified, of which 143 (92%) were assigned the correct subclass. Next, using the SSP to classify the 527 ER^+ ^samples into the intrinsic subtypes, we then assigned each of the 527 ER^+ ^samples into an ER^- ^subclass based on the same nearest centroid criterion (Table 3). As expected, only 4% of ER^+ ^tumors were classified as basal, whereas the majority (82%) of them were luminal. Moreover, the analysis showed that ER^+ ^luminal B samples were most similar to CC^+ ^and CC^+^/IR^+ ^ER^- ^samples, which is consistent with the fact that all of these samples over-express cell cycle and cell proliferation genes. In contrast, ER^+ ^luminal A samples were most similar to ECM^+ ^(63%), IR^+ ^(26%), and SR^+ ^(8%) ER^- ^samples. Not surprisingly, almost all (16/19 [84%]) 'normal' ER^+ ^samples were most similar to ECM^+ ^ER^- ^samples. All basal ER^+ ^samples had expression profiles most similar to CC^+^/IR^+ ^and CC^+ ^subtypes. Interestingly, only 16 of the 42 (38%) ER^+ ^HER2^+ ^tumors exhibited significantly correlated expression profiles to any one of the five ER^- ^subclasses, with most of these (11) mapping to the CC^+^/IR^+ ^subtype.

**Table 3:** Classification of ER^+ ^intrinsic subtypes, medullary breast cancer, and BRCA1 tumors into ER^- ^subclasses

	ER^+^					MBC	DBC	BRCA1
				
	Luminal B	Luminal A	Normal	Basal	HER2			
*n*	97	337	32	19	42	22	44	18
*n *classifiable	37	113	19	17	16	20	33	16
CC^+^/IR^+^	17	2	0	13	11	14	4	13
CC^+^	20	2	0	4	1	3	3	2
ECM^+^	0	71	16	0	0	1	3	1
SR^+^	0	9	0	0	0	0	20	0
IR^+^	0	29	3	0	4	2	3	0

Classification of ER^+ ^intrinsic subtypes (527 samples from the integrated cohort NKI2 + EMC + NCH), 22 medullary breast cancers (MBCs) and 44 ductal breast cancers (DBCs) from [38], and 18 BRCA1 mutants from [3] into ER^- ^subclasses, using the nearest centroid criterion with Pearson correlation as distance metric. Only samples with Pearson correlation coefficients larger than 0.25 were deemed classifiable. ER, estrogen receptor.

### A subgroup of good prognosis in ER^- ^breast cancer

We next considered whether the five identified clusters were associated with different prognostic groups. Because we had merged different cohorts, and it is questionable whether survival data can be also merged together, we decided to test first the clusters for association with clinical outcome by using a dichotomized outcome variable. Specifically, poor outcome was defined as any death or distant metastasis event, whereas good outcome was defined as a patient alive or with no distant metastasis. By studying the distribution of good and poor outcome events in the respective clusters, significant associations with prognosis could be found by means of Fisher's exact test (Table 2). Interestingly, this showed that the IR^+ ^subgroup had better prognosis when compared with the ECM^+ ^(*P *= 0.08), CC^+ ^(*P *= 0.03), and SR^+ ^(*P *= 0.005) subclasses.

Compared with the CC^+^/IR^+ ^subgroup it also had better prognosis, although the difference was not statistically significant (*P *= 0.19). We thus combined the CC^+^/IR^+ ^and IR^+ ^subclasses together and evaluated the prognosis of this larger subclass relative to the rest of ER^- ^samples. Consistent with our previous result [17] we found that the ER^- ^tumors over-expressing immune response genes had better prognosis than ER^- ^samples that did not (*P *= 0.02).

To further confirm our findings, we also generated Kaplan-Meier survival curves for the five identified subclasses using TTDM as the end-point (Figure 3b). This showed that the SR^+ ^and CC^+ ^subclasses had worst prognosis, whereas the IR^+ ^subclass was the group with best prognosis. Specifically, relative to the IR^+ ^subclass the SR^+ ^subgroup had a hazard ratio (HR) of 3.70 (95% confidence interval [CI] 1.49 to 9.24; *P *= 0.005), whereas the CC^+ ^subgroup had an HR of 2.75 (95% CI 1.07 to 7.05; *P *= 0.035). Similarly, relative to the CC^+^/IR^+ ^subclass, the SR^+ ^subgroup had an HR of 2.35 (95% CI 1.13 to 4.88; *P *= 0.02), whereas for the CC^+ ^subgroup it did not quite reach statistical significance (HR 1.80, 95% CI 0.83 to 3.89; *P *= 0.13). We verified the statistical significance of these survival differences by 10,000 random permutations of the samples, which showed that the theoretical *P *value estimates above were essentially identical to the empirically derived *P *values.

### Prognostic markers in ER^- ^tumors are associated with immune response functions

**Figure 4:**
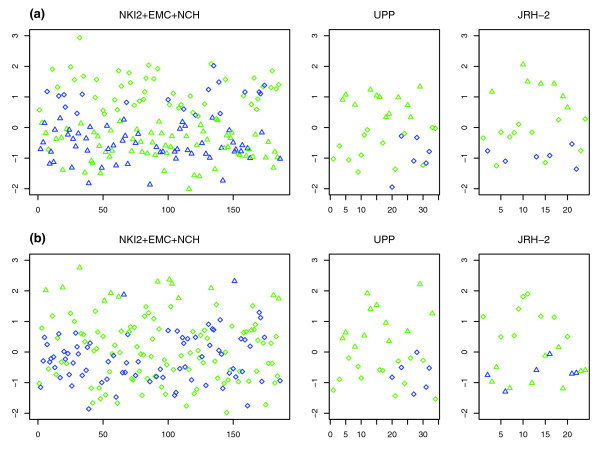
Expression profiles of selected prognostic markers in ER^- ^breast cancer. Expression profile (on a log2 scale) of selected prognostic markers **(a) ***IGLC2 *and **(b) ***C1QA *in the integrated cohort of 186 ER^- ^tumours (NKI2 + EMC + NCH), and in the validation cohorts UPP and JRH-2. Good outcome samples are shown in green, and poor outcome samples in blue. Clusters were inferred using the variational Bayesian approach in NKI2 + EMC + NCH and the pam algorithm in the UPP and JRH-2 cohorts. Infered clusters are indicated by different shapes (triangles and diamonds). ER, estrogen receptor; pam, partitioning around medoids.

The better prognosis of the IR^+ ^subclass is likely due to specific genes that are individually prognostic. In order to find these, we applied the PAC algorithm to the 813 genes with negative kurtosis expression profiles. Briefly, this procedure selects features based on whether their expression profiles are best described as a mixture of gaussians or not, and then evaluates whether the distribution of good and poor outcome events among the inferred clusters is statistically different from random or not. Applying PAC, we found 22 genes with a Fisher test *P *< 0.05 (Additional data file 1). A Gene Ontology (GO) enrichment analysis of these 22 genes using the GOTree Machine [28] showed that immune/defense response was the most enriched GO, with seven genes falling into this category (*C1QA*, *IGLC2*, *LY9*, *TNFRSF17*, *SPP1*, *XCL2*, and *HLA-F*). The expression profiles for these seven genes confirmed the presence of distinct clusters with nonrandom distributions of good and poor outcome samples (Figure 4 provides detailed profiles of *C1QA *and *IGLC2*).

In contrast, application of the same method to ER^+ ^breast cancer yielded 29 genes (out of a possible 193) with *P *< 0.05 (Additional data file 2). In spite of there being only 29 genes in this list, there were many with mitotic cell cycle functions, notably *UBE2C*, *MAD2L1*, *E2F1 *and *KIFC1*, and GO enrichment analysis confirmed that the cell cycle GO was the most significantly enriched category followed by transcription regulatory activity. This result for ER^+ ^breast cancer confirms findings reported elsewhere that poor prognosis in ER^+ ^breast cancer is related mainly to over-expression of genes in cell cycle and cell proliferation pathways [3,5,7]. Notably, none of the identified prognostic genes were related to immune response functions.

Having identified a prognostic module of seven immune response genes (henceforth called the IR module), we next confirmed that clustering over this module resulted in clusters significantly associated with clinical outcome. Specifically, the partitioning around medoids (pam) clustering algorithm [29] with two centers predicted one cluster with 52 good outcome and 56 poor outcome patients, and another cluster with 56 good outcome and only 19 poor outcome events, which was highly significant under Fisher's exact test (*P *= 0.0004; Figure 3c). Kaplan-Meier and Cox regression analyses further confirmed that the group not over-expressing the seven-gene module (out of the seven genes the majority [six] are not over-expressed) had a greater risk for distant metastasis (HR 2.02, 95% CI 1.2 to 3.4; *P *= 0.009; Figure 3d).

### Relation to *STAT1 *and IFN cluster

Next, we investigated the relation of the good prognosis subgroup identified here with the novel IFN regulated cluster identified recently [23]. As shown previously [23], the IFN cluster, defined by over-expression of interferon regulated genes, including the transcriptional regulator *STAT1*, was most closely related to the basal subclass and had a prognostic performance similar to that of luminal B samples when compared with the luminal A and normal subtypes. Interestingly, when compared with the basal and HER2^+ ^subclasses, the IFN group had better prognosis, although a formal comparison was not conducted by Hu and coworkers [23].

Among the 98 genes in the immune response gene cluster (Figure 3a and Additional data file 1), we identified a total of 14 with interferon related functions, including *STAT1*, *SP110*, *NFKBI*, *IFI44*, *IFNGR1*, *ISGF3G*, and *IRF7*. Interestingly, however, none of these genes showed association with prognosis except *SP110*. Thus, although it appears that our subclass is related to the IFN class discovered by Hu and coworkers [23], it is also distinct in that the associated prognostic markers are not in the IFN cluster.

### Immune response module predicts outcome independently of lymph node status

Because our IR gene cluster included *STAT1 *and interferon-induced genes, and these genes have been shown to be associated with lymph node (LN) metastasis [30], we considered whether the subgroup of ER^- ^samples over-expressing the 98-gene IR cluster was significantly associated with LN status. Because all patients in the ECM cohort were LN negative, this analysis was only performed on the NKI2 and NCH cohorts (109 ER^- ^patients, of whom 42 had LN involvement). Using pam clustering with two centers over these 98 genes and 109 samples, we found subgroups with similar proportions of LN metastases (43 LN negative and 28 LN positive versus 24 LN negative and 14 LN positive; Fisher's exact test *P *= 0.84). Moreover, clustering only over the 14 genes involved in the IFN subcluster still did not show a significantly nonrandom distribution of LN metastases among the clusters (*P *= 0.23), although in agreement with Huang and coworkers [30] the cluster over-expressing the IFN genes had proportionally more LN metastases. Similarly, the distribution of LN metastases among the two clusters predicted by the IR module was not significantly different from random (*P *= 0.48). While LN status itself was a significant predictor of distant metastasis both in univariate (HR 2.28, 95% CI 1.36 to 3.85; *P *= 0.002) and multivariate (HR 2.16, 95% CI 1.28 to 3.64; *P *= 0.004) Cox regression analysis (this result is for a multivariate model including LN status and the IR module as predictors.), importantly, the IR module remained a prognostic predictor for TTDM (HR 1.93, 95% CI 1.14 to 3.26; *P *= 0.015) in the multivariate model that included LN status (Table 4). This showed that the IR module identified here adds independent prognostic value over LN status in ER^- ^breast cancer.

**Table 4:** Univariate and Multivariate Cox-regression model

	ER^- ^(*n *= 186)		ER^+ ^(*n *= 527)	
	HR (95% CI)	*P*	HR (95% CI)	*P*

LN	2.28 (1.36-3.85)	0.002	2.07 (1.47-2.90)	<10^-4^
LI	1.06 (0.66-1.70)	0.9	1.50 (0.72-3.14)	0.58
IRM	2.02 (1.19-3.41)	0.009	1.25 (0.91-1.71)	0.19
LN^a ^+ IRM	2.16 (1.28-3.64)	0.004	2.10 (1.49-2.96)	<10^-4^
LN + IRM^a^	1.93 (1.14-3.26)	0.015	1.29 (0.94-1.76)	0.11
LI^a ^+ IRM	0.86 (0.32-2.28)	0.76	1.75 (0.41-7.47)	0.45
LI + IRM^a^	2.05 (0.71-5.97)	0.19	0.57 (0.27-1.19)	0.13
LN^a ^+ LI + IRM	1.79 (0.70-4.62)	0.22	1.48 (0.68-3.19)	0.32
LN + LI^a ^+ IRM	0.84 (0.51-1.38)	0.72	1.65 (0.38-7.07)	0.5
LN + LI + IRM^a^	2.22 (0.76-6.50)	0.15	0.57 (0.27-1.20)	0.14

The table summarizes the hazard ratio (HR), 95% confidence interval (CI), and log-rank test *P *values of univariate Cox proportional hazards regression models, with lymph node status (LN = 1/0 for LN +/-), level of lymphocytic infiltration (LI = 1 for low infiltration score, and LI = 0 for high infiltration score) and the classification based on the seven-gene immune response related module (IRM; 2 = down-regulation of module, 1 = upregulation of module) as predictors. ^a^Corresponding values in the multivariate Cox models including LN, LI, and IR module as predictors. The table compares the values for estrogen receptor (ER)^- ^and ER^+ ^breast cancer.

### Immune response module predicts outcome independently of lymphocytic infiltration

The upregulation of immune response genes in good prognosis tumors could be explained by the fact that these tumors elicit a stronger immune and inflammatory response, as measured for example by a higher degree of lymphocytic infiltration (LI). The association of high LI with good prognosis is well known [31-34], and although a few conflicting results have also been reported [35-37], we thought it natural to consider whether upregulation of the identified immune response module conferred good prognosis independently of LI. To this end, we scored the samples in the NCH cohort for LI (see Materials and methods, below) and combined these with the available LI score information from the NKI2 cohort, yielding a total of 50 scored samples. We found that although there were proportionally more tumors with high LI scores in the group over-expressing the immune response genes (specifically there were 11 high LI and 9 low LI samples versus 8 high LI and 22 low LI in the under-expressed group; Fisher test, *P *= 0.07), a multivariate Cox regression with TTDM as the outcome variable and the seven-gene IR module and LI score as predictors showed that the immune response module was still a strong predictor of clinical outcome, independent of LI (HR 2.05, 95% CI 0.71 to 5.97; *P *= 0.19; Table 4). (The *P *value is only marginally below statistical significance, which is most likely due to the relatively small sample size.) Supporting this result further, we did not find in ER^- ^tumors any significant association between LI and clinical outcome (Table 4).

### Relation to medullary and BRCA1 ER^- ^breast cancer

To further confirm the independent prognostic power of the IR module from LI, we investigated the relationship of the identified good prognosis tumors with medullary breast cancers (MBCs), which are characterized by high LI scores and relatively good prognosis [38]. Thus, we considered to which ER^- ^subclass the 22 MBC expression profiles from the report by Bertucci and coworkers [38] were most similar (Table 3). (For completeness and reference, we also performed the analysis for the 44 ductal breast cancers [DBCs], also profiled by Bertucci and coworkers [38].) This showed that of the 22 MBCs, 20 showed reasonably strong correlation to one of the ER^- ^subclass centroids, 14 of which (70%) were most similar to the CC^+^/IR^+ ^subtype, whereas only two (11%) were most similar to the IR^+ ^subtype. In contrast, for the 33 DBC which could be classified, only four (12%) and three (9%) were most similar to the CC^+^/IR^+ ^and IR^+ ^subtypes. These results mirror the distribution of LI scores across the five ER^- ^subclasses (Figure 3a and Table 2) and further confirms that the best prognostic group (IR^+^) is not related to MBC, whereas CC^+^/IR^+ ^is.

A similar analysis was performed for BRCA1 mutant tumors. Of the 16 BRCA1 mutants from the NKI2 cohort [3], which were also deemed classifiable based on a 0.25 correlation threshold, 13 (81%) had expression profiles most similar to the CC^+^/IR^+ ^subtype (Table 3). None showed similarity to the IR^+ ^subclass. Therefore, this suggests that ER^- ^BRCA1 mutants, in common with MBCs, are most similar to the CC^+^/IR^+ ^subclass identified here.

### External validation

Having identified a prognostic module related to immune response, we next attempted to validate this finding using two independent breast cancer cohorts [7,19]. Specifically, the hypothesis to be tested was that over-expression of the identified prognostic markers is associated with good prognosis, except for *SPP1*, for which good prognosis is hypothesized to be associated with under-expression. Because of the relatively small sample size of the two external cohorts, an algorithm that attempts to learn the optimal number of clusters (as implemented in PACK) is unlikely to capture structure because of a large false negative rate [39]. Hence, in order to define groups of over-expression and under-expression, we applied the pam algorithm with two centers to each of the seven genes in each of the two cohorts (Figure 4 and Additional data files 6 and 7). Because the small number of events, six, in each of the two external cohorts implied a highly discrete *P *value distribution, Fisher test *P *values would be too conservative and poor approximations for type I errors [40]. To overcome this difficulty, we also considered the distribution of good and poor outcome samples over the combined clusters of over-expression and under-expression (Table 5). This showed that four of the seven genes (*C1QA*, *IGLC2*, *TNFRSF17*, and *LY9*) were also highly prognostic in these two external cohorts, thus confirming the validity of our finding. Moreover, as in the three original cohorts, over-expression of these genes in the two external cohorts was associated with good prognosis (Figure 4 and Additional data files 6 and 7). For the other three genes (*HLA-F*, *SPP1*, and *XCL2*), *P *values did not reach statistical significance (*P *about 0.2), yet their expression profile trends were entirely consistent with those found in the integrated cohort, thus confirming their role as members of a robust prognostic module (Additional data files 6 and 7).

**Table 5:** External validation of immune response prognostic module: distribution of poor and good outcome patients in over-and-underexpressed subgroups

Gene		UPP			JRH-2			Combined		
		
		Poor	Good	*P*	Poor	Good	*P*	Poor	Good	*P*
IGLC2	High	0	12	0.04	0	7	0.09	0	19	0.003
	Low	6	13		6	11		12	24	
LY9	High	1	16	0.05	0	6	0.14	1	22	0.007
	Low	5	9		6	12		11	21	
TNFRSF17	High	1	16	0.05	0	10	0.02	1	26	0.001
	Low	5	9		6	8		11	17	
C1QA	High	0	12	0.04	0	9	0.04	0	21	0.001
	Low	6	13		6	9		12	22	

**Figure 5:**
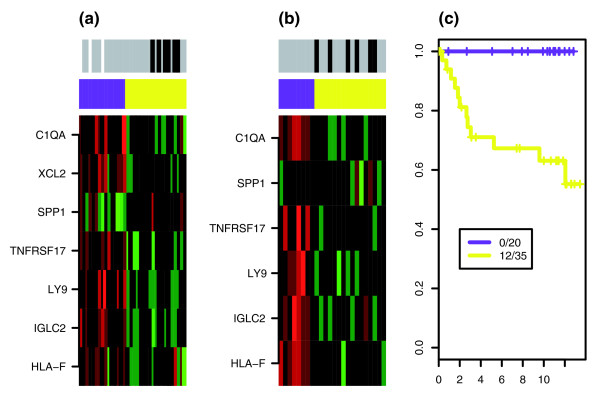
Pam clustering over IR module in external ER^- ^cohorts. Heatmap of gene expression of seven-gene IR-module in ER^- ^samples of the **(a) **UPP and **(b) **JRH-2 cohorts. Shown are the clusters over-expressing (purple) and under-expressing (yellow) the IR module, as predicted by the pam algorithm. Good outcome samples are shown in gray, and poor outcome samples in black. Green indicates relative under-expression, and red indicates relative over-expression. **(c) **Kaplan-Meier survival curves over combined external cohorts (for UPP end-point was disease-specific survival, and for JRH-2 it was recurrence-free survival), with the number of events and samples in each of the two predicted groups. ER, estrogen receptor; pam, partitioning around medoids.

To confirm the robustness of our findings, we used the pam algorithm to classify patients in the two external cohorts into clusters over-expressing and under-expressing the IR module (Figure 5a,b). Remarkably, the predicted 20-sample cluster over-expressing the module was composed entirely of good outcome patients, whereas the remaining 35-sample cluster included 12 poor outcome events (Figure 5c), which was highly significant under Fisher's exact test (*P *= 0.002; the HR is singular because one cluster did not have any events).

### The prognostic value of the IR module is specific to ER^- ^tumours

To confirm that the good prognosis conferred by activation of the IR module is specific to ER^- ^breast cancer, we applied the same pam clustering algorithm over the seven genes to the integrated dataset of 527 ER^+ ^breast tumors. This gave two clusters with unequal distributions of good and poor outcome samples (209 good and 99 poor for the cluster under-expressing the genes versus 163 good and 47 poor for the cluster over-expressing the genes; *P *= 0.02). Although this suggested to us that over-expression of this seven-gene module also conferred better prognosis in ER^+ ^samples, the association was much weaker than for ER^- ^samples. Univariate Cox regression with TTDM as the outcome variable confirmed that under-expression of this seven-gene module conferred a much greater risk for distant metastasis in ER^- ^tumors (HR 2.02, 95% CI 1.2 to 3.4; *P *= 0.009) than in ER^+ ^tumors (HR 1.25, 95% CI 0.9 to 1.7; *P *= 0.16; Table 4). It is also noteworthy that, in contrast to the ER^- ^case, in the multivariate model setting for ER^+ ^tumors, a low LI score and LN involvement were stronger predictors of TTDM than the seven-gene module (HR 1.65, 95% CI 0.4 to 7.1 for LI score and HR 1.48, 95% CI 0.7 to 3.2 for LN involvement, versus HR <1; Table 4). The specificity of our prognostic module to ER^- ^breast cancer was confirmed by application of PAC, which showed that, with the exception of *XCL2*, none of the other six genes were individually prognostic.

In this context, it is worth noting once again the absence of immune response related genes among the 29 PAC derived prognostic genes in ER^+ ^disease, which would suggest that a good prognosis IR related subtype is absent in ER^+ ^breast cancer. To investigate this further we checked, by performing Wilcoxon rank sum tests on the 5,007 genes, that the absence of immune response GOs was not just an artefact of the small number of genes picked out by PACK. In fact, GO analysis (using GOTree Machine) of the top 500 genes obtained from this rank sum test (all with q-values < 0.05) showed that cell cycle and transcription regulator activity related GOs were the only categories with highly significant *P *values (uncorrected *P *< 10^-6^), and that the only enriched immune response related GO was that of humoral immune response, with 11 represented genes (including *BLM*, *FADD*, *C3*, *C7*, *BCL2*, *NFKB1*, and the IR module member *TNFRSF17*), and which was only marginally enriched (uncorrected *P *= 0.005). Although we verified that this humoral immune response module was associated with prognosis in the integrated ER^+ ^cohort independent of LN status and LI (HR 2.26, 95% CI 0.97 to 5.26; *P *= 0.06) we were unable to validate this prognostic module in the external UPP and JRH-2 cohorts (Additional data file 8). Moreover, the co-regulation patterns for the genes in this module were less coherent than those for the IR module in ER^- ^breast cancer (Figure 5 and Additional data file 8). Hence, independent of the methodology used, an IR related prognostic module in ER^+ ^breast cancer could not be identified, which seems to suggest that a good prognosis subtype related to IR is specific to ER^- ^disease.

## Discussion

A striking difference between ER^+ ^and ER^- ^disease is emerging at the level of mRNA expression. Although in ER^+ ^disease a significant number of genes have been found that correlate with clinical outcome [5,10,18,22], in ER^- ^disease no such prognostic signatures have thus far been reported. Moreover, although in ER^+ ^tumors subtypes of different prognostic risks, the luminal A and B subtypes, have been defined [21,22], no such subdivisions have been noted for ER^- ^breast cancer. It is known that the two main subtypes of ER^- ^breast cancer (ER^-^/HER2^+ ^and basals) have worse prognosis compared with the luminal A subtype, but no outcome differences between the ER^-^/HER2^+ ^and basal subtypes have been observed [15,21-23,26].

We believe that these differences between ER^+ ^and ER^- ^disease are related to the different histopathologic characteristics of the tumors. The prognostic signatures derived for ER^+ ^breast cancer are characterized by genes related to cell cycle and cell proliferation pathways, and are also highly correlated with the histologic grade of the tumors [7,22]. It is not a coincidence that most luminal B tumours are of high grade, whereas the great majority of luminal A tumours are of low grade [7,22]. It appears that there may be a whole plethora of diverse oncogenic pathways that drive the over-activation of cell cycle and cell growth pathways in poor prognosis tumors. This would explain the larger number of prognostic genes found in ER^+ ^disease (the great majority of which are related to cell cycle ontologies) as well as the stronger prognostic signals (relatively large differences in log2 expression between poor and good prognosis tumours), an effect that is probably driven by oncogenic amplifications. This interpretation would also fit in well with our finding that most bimodal profiles in ER^+ ^breast cancer have positive kurtosis values, because this could be a reflection of a more diverse range of small amplifier subgroups in ER^+ ^breast cancer.

In contrast, most ER^- ^tumours are of high grade, which would explain why any differences in clinical outcome within ER^- ^disease are not related to differential activation of cell cycle pathways. Instead, the work presented here shows that differences in clinical outcome within ER^- ^disease are mainly related to differentially expressed genes in the complement and immune response pathways, and that the association with prognosis can be independent of lymphocytic infiltration (LI) and LN status. In fact, for ER^- ^tumors we observed that even though there were proportionally more high LI samples in the group over-expressing the IR module, these did not necessarily have better prognosis. The fact that LI could not explain the observed association of the IR module with outcome was supported further by our finding that medullary breast cancers, which are characterized by high LI scores, had expression profiles most similar to the CC^+^/IR^+ ^subtype rather than the IR^+ ^subtype, which had the best prognosis overall. The better prognosis of the CC^+^/IR^+ ^subtype relative to CC^+ ^and SR^+ ^ER^- ^breast cancer is therefore entirely consistent with the CC^+^/IR^+ ^subclass being medullary breast cancers (MBCs), as MBC is known to have marginally better prognosis than other basal tumors [38]. On the other hand, the IR^+ ^subclass, which had the best prognosis among the five ER^- ^subclasses, was only marginally associated with high LI and was unrelated to MBC. Also consistent with these observations, it is important to note again the distinction between the identified seven-gene prognostic IR module and the 98-gene IR cluster that was derived from unsupervised hierarchical clustering. Clearly, we found a strong statistical association between high LI and over-expression of the 98-gene IR cluster. Specifically, there were 13 high LI and eight low LI samples in the combined IR^+ ^and CC^+^/IR^+ ^clusters relative to six high LI and 23 low LI samples in the rest of the cohort (Fisher test *P *= 0.007). In contrast, the association between high LI and over-expression of the IR module was much weaker (*P *= 0.07). Again, this suggests that a significant number of genes in the 98-gene IR cluster show expression variability that is not explained by LI. This is confirmed further by two recent studies that profiled breast cancer cell lines [27,38], which showed that a considerable number of immune response related genes do exhibit significant variable expression across the basal cell subtype. Moreover, we found that two (*SPP1 *and *HLA-F*) of the three IR module genes that we could map to good quality probes in [27,38] showed twofold changes across the eight basal cell lines.

Thus, these findings together suggest that a significant proportion of the expression of the IR module genes in the good prognosis tumors is tumor-intrinsic in origin. That tumor-intrinsic expression of IR genes can have an impact on prognosis of breast cancer patients is plausible in view of recent studies that show, for example, how amplification of kinase oncogenes can activate the nuclear factor-*κ*B pathway, and hence immune response pathways, in both breast cancer cell lines and patient derived breast tumors [41]. Similarly, another recent study [42] used breast cancer cell lines to show how BRCA1/IFN-*γ *pathways may regulate target genes involved in innate immune response, providing another possible mechanism for tumor intrinsic IR gene expression variability.

In spite of identifying only a relatively small module of prognostic genes, we were nevertheless able to validate their prognostic potential in two external cohorts. It is likely that an integrative analysis similar to the one used here but applied to multiple cohorts that were all profiled on exactly the same genome-wide platform would allow further expansion of this module to include other members of the complement and immune response pathways. Interestingly, from the seven prognostic markers that composed the IR module, two have already been associated with clinical outcome in breast cancer. Specifically, *C1QA*, which is a gene involved in the classical complement pathway, was recently shown to harbor a single nucleotide polymorphism that correlated with distant metastasis in breast cancer [43]. Two recent studies also implicated *SPP1 *(osteopontin) in metastatic breast cancer [44,45].

It is important also to note that the robustness of the identified prognostic markers is a consequence of the PACK methodology. Despite being a conservative procedure that filters out many true positives, PACK allows, by efficient removal of false positives, a more reliable identification of prognostic markers. We tested this further by applying two popular statistical tools, singular value decomposition (SVD) [46] and the shrunken centroids classifier (PAMR) [47], to the integrated ER^- ^dataset to determine whether we could derive a similar if not identical prognostic IR module. Using SVD we found that none of the inferred SVD components showed a correlation with prognosis (Wilcoxon rank sum test *P *> 0.05), whereas at a FDR threshold of 0.3 PAMR yielded 21 prognostic genes, of which only two (the IR module member *TNFRSF17 *and *KLRD1*) had immune response related functions. Thus, in agreement with findings presented previously [17], this reinforces the advantage of PACK over other pattern recognition tools and supervised methods that do not use pattern recognition steps, such as for example those based on *t*-tests.

The absence of a prognostic IR module in ER^+ ^breast cancer is intriguing. The seven-gene IR module was only marginally associated with prognosis in ER^+ ^disease, and importantly this association was not independent of LI or LN status. Repeating the same unsupervised analysis (PAK) and semi-supervised analysis (PACK) in ER^+ ^breast cancer also did not find a prognostic immune response module. By using a traditional supervised method, a prognostic IR module was identifiable but failed to validate in the two external cohorts. Thus, to determine fully whether such a robust prognostic IR module exists for ER^+ ^breast cancer, it may be necessary to conduct larger integrative studies that use the same microarray platform so that the analysis can be performed over a larger set of common genes.

Besides identifying a module of genes that is prognostic in over 240 ER^- ^breast tumors, PACK also provided us with a novel subclassification of ER^- ^breast cancer. Specifically, clustering over PACK selected genes identified five different subtypes (CC^+^, CC^+^/IR^+^, IR^+^, ECM^+^, and SR^+^) characterized by the over-expression patterns of four distinct gene clusters, each enriched for IR, ECM, CC, and SR genes, respectively. Moreover, we related these subtypes to the gene expression based intrinsic subclasses. This showed that the basal subgroup was a heterogeneous group with at least four distinct subtypes (CC^+^, CC^+^/IR^+^, ECM^+^, and IR^+^), whereas the ER^-^/HER2^+ ^subgroup showed strong overlap with the SR^+ ^and IR^+ ^subtypes.

## Conclusion

While in ER^+ ^breast cancer prognostic markers are associated mainly with cell cycle pathways, in ER^- ^disease prognostic markers are associated with immune response pathways. In particular, we have identified a subclass of ER^- ^tumors that over-express immune response genes and that has a good prognosis compared with the rest of ER^- ^breast tumors, independently of LN status or LI. Furthermore, we have identified an associated module of complement and immune response genes that define prognostic markers valid in over 240 ER^- ^samples.

## Materials and methods

### Datasets and gene annotation

The microarray breast cancer datasets considered in this work are described elsewhere [5,7,9,18,19]. For these cohorts we used the normalized data, which are available in the public domain (see [5,7,9,18,19]). The retrieved datasets were further normalized, if necessary, by transforming them onto a common log2 scale and shifting the median of each array to zero. We also created an automated computational pipeline (Perl scripts on a Linux platform) to crosslink the annotation provided for each dataset with UniGene. For some datasets, the linkage relied on Ensembl [48] external database identifiers. Thus each probe was associated with a universal gene name. This procedure generated a nonredundant set of gene identifiers for the subsequent integrative analysis.

### PACK: profile analysis using clustering and kurtosis

The hypothesis underlying PACK [17] is that genes that are true biologic or clinical markers have expression profiles that are generated by a mixture of two or more underlying distributions, whereas spurious features are more likely to have profiles generated by a single distribution. The biologic validity of this assumption was proved through a FDR analysis [17].

PACK can be viewed as a semi-supervised algorithm, consisting of two main steps: a feature selection criterion and a supervised step, in which the selected features are correlated to a phenotype (Figure 2). It is important to note that PACK is a flexible modular algorithm in that the feature selection step can be applied on its own. In this case, there are two possible versions of the algorithm: PAC and PAK. The precise way in which these two algorithms are used in PACK will depend on the purpose of the exercise. Below, we describe the PACK strategy implemented in this paper, which is slightly different from that applied previously [17].

### Feature selection with PAK: using negative kurtosis to find genes defining major subclasses

Kurtosis is related to the fourth central moment and can conveniently be defined as follows [49]:

$$K(X)\equiv \frac{E[{\left(X-\bar{X}\right)}^{4}]}{E{\left[{\left(X-\bar{X}\right)}^{2}\right]}^{2}}-3$$

where X is any random variable and E denotes the expectation. For a gaussian $E[{\left(X-\bar{X}\right)}^{4}]=3E{\left[{\left(X-\bar{X}\right)}^{2}\right]}^{2}$, so that K(X) = 0. Most nongaussian distributions necessarily have either K > 0, in which case they are called supergaussian or leptokurtic, or K < 0, in which case they are called subgaussian or platykurtic. Specifically, a mixture of two approximately equal mass gaussians must have negative kurtosis because the two modes on either side of the center of mass effectively flatten out the distribution. To see this, consider a gene whose expression profile is described by a mixture of two gaussians. Then, the kurtosis, K, is a function of two parameters (we assume for simplicity that the gaussians are of equal variance *σ*^2^, although this assumption is not needed for the result below); the effect size of the gene, as defined by the effective separation e between the two gaussians (e = *μ*/*σ*, where *μ *is the separation), and the ratio of cluster weights (*π*_1_, *π*_2_), that is R = *π*_1_/(1 - *π*_1_). Specifically, a short computation reveals that

$$K(e,R)=e^{4}\frac{R(R-a)(R-b)}{{\left(1+R\right)}^{4}{\left(1+\frac{R}{{\left(1+R\right)}^{2}}e^{2}\right)}^{2}}$$

where a and b are the quadratic roots 2 ± √3. Thus, for e ≠ 0, the kurtosis is negative if and only if (2 - √3) < R < (2 + √3). This in turn requires the smallest cluster weight, *π*_min_, to be in the range (approximately) of 0.22 <*π*_min _< 0.5. It follows that for the case of approximately equal weights, where R ≅ 1 (*π*_min _≅ 0.5), the kurtosis is always negative and in the limit of large cluster separations (when e >> 1) the kurtosis decreases monotonically, asymptotically approaching the lower bound -2. Thus, kurtosis provides a useful measure for ranking genes based on how platykurtic their profiles are.

Given a gene's expression profile x = (x_1_, ..., x_n_), an unbiased estimate for the kurtosis [50] is as follows:

$$K(x)≙\frac{n(n+1)\sum_{i=1}^{n}{\left(x_{i}-\bar{x}\right)}^{4}}{(n-1)(n-2)(n-3)\sigma^{4}}-\frac{3{\left(n-1\right)}^{2}}{\left(n-2)(n-3\right)}$$

where $\bar{x}$ and *σ *are the mean and standard deviation estimates of the profile. A standard error estimate of K was obtained by performing 10,000 random simulations, with *n *= 186 (number of ER^- ^samples), which showed that the standard error estimate, 0.36, was essentially identical to the theoretical estimate, √(24/*n*) [50].

Two notes with the feature selection step are in order. First, the kurtosis threshold used to select features depends on how large the smallest subgroup must be. Generally, given the effective separation values that are typical for differential gene expression, we find that a zero kurtosis threshold (as used in this report) generally picks out subgroups within the individual gene expression profiles that are at least as large as 30% of the total sample size [17]. Second, in principle, genes defining major subclasses could be found using a clustering step to infer two clusters (PAC) and setting a lower bound threshold (for instance, 30%) on the size of the smallest cluster. However, this approach is computationally more expensive, because PAC attempts to estimate the optimal number of clusters in the profile. However, this model selection step is a necessary one to ensure that profiles for which there is no objective evidence of bimodality are excluded (see below).

### PAC: identification of robust prognostic markers

Having selected the genes defining the largest subclasses, we next apply PAC to each of these genes to remove those for which there is no evidence of bimodality (gaussian profiles that spuriously have negative kurtosis values). Specifically, given a gene's expression profile x = (x_1_, ..., x_n_), we model this as a random sample of a univariate random variable X, whose density function is possibly a mixture of Gaussians:

$$p(x_{i}|\theta )=\sum_{k=1}^{C_{M}}\pi_{k}G(x_{i}|\mu_{k},\sigma_{k})$$

Where *π*_k _are the weights of the components, (*μ*_k_, *σ*_k_) are the mean and standard deviation of the univariate gaussian k, and *θ *denotes the set of all parameters. In the above, C_M _denotes the maximum number of clusters that can be inferred, which in our application we set to 2. The optimal number of clusters, C, can be inferred using one of various approaches. One possibility is to use the EM algorithm to learn the parameters for the two different models C = 1 and C = 2, and perform model selection using the Bayesian Information Criterion (BIC) score [51,52]. Alternatively, the optimal number of clusters, C, can be inferred using a lower bound on the model evidence, as provided by a variational Bayesian (VB) approach [39,53,54]. The results we report here were obtained using the VB algorithm for model selection. Thus, genes for which C = 1 were excluded from further analysis. Finally, association with the phenotype (here prognosis) was determined using Fisher's exact test to test whether poor outcome events were unevenly distributed across the two clusters.

### Software packages used

All analyses were performed using the R statistical programming language [55]. The following add-on packages were used: vabayelMix for the PACK implementation, survival for the Cox regression models, qvalue for FDR estimation, and cluster for the partitioning around medoids (pam) clustering algorithm.

### The SSP classifier

The classification of the samples in the NKI2, EMC, and NCH cohorts into the intrinsic subtypes was performed using the single sample predictor (SSP) [23] and was done for each cohort separately because this guaranteed a larger number of overlapping genes. In the SSP, samples were assigned the intrinsic subtype for which the corresponding Spearman rank correlation between the sample and SSP centroid was maximal [23].

### The ER^- ^subclass centroids

From the hierarchical clustering with Pearson correlation metric and complete linkage diagram (Figure 3a) we constructed mean centroids for each of the five subclasses. Classification of external samples to these centroids was performed using the nearest centroid criterion. Because these centroids were defined over ER^- ^samples only, external samples (which may not be ER^-^) may not show strong correlation to any of these centroids. We thus validated, through 10,000 Monte Carlo (MC) randomisations, that samples with a maximal pearson coefficient larger than 0.25 were significantly correlated with the corresponding centroid (*P *< 0.0001). Samples with maximal correlation coefficients smaller than 0.25 were deemed to be unclassifiable.

### Expression based basal and HER2^+ ^markers

The basal marker used in Figure 3 was derived by first mapping ten validated basal markers (*CRYAB*, *ANXA8*, *LAMC2*, *LAMB3*, *ITGA6*, *KRT17*, *KRT15*, *KRT13*, *KRT6B*, and *KRT5*) [27] onto the integrated data set of 5,007 genes. For each of these markers samples were ranked in order of decreasing expression or 'basalness'. For each sample in the integrated cohort an average rank was then computed over the ten basal markers. The average ranks were then rescaled onto the unit interval (0,1), with '1' indicating highest expression for basal markers. The marker for the *ERBB2 *subtype was obtained in an analogous manner using three genes in the *ERBB2 *amplicon (*ERBB2*, *GRB7*, and *STARD3*).

### Lymphocyte infiltration scores

For the samples from our NCH cohort [9] we used the following scoring method. Lymphocytic infiltration (LI) was assessed in whole tumour sections from frozen sections stained with hematoxylin and eosin. The intensity of lymphocytic infiltrate was first graded semi-quantitatively as minimal or mild (1), moderate (2), and marked (3). The LI scores were then dichotomized (we considered mild and moderate as low LI and marked as high LI) to make them comparable with the binary LI scores used by van 't Veer and coworkers [3].

## Additional data files

The following additional data are available with the online version of this manuscript. Additional data file 1 is a table showing the 813 genes with negative kurtosis expression profiles over 186 ER- tumors, together with the predicted number of clusters and Fisher's test P value with outcome as binary phenotype. Additional data file 2 is a table showing the 193 genes with negative kurtosis expression profiles over 527 ER+ samples, together with the predicted number of clusters and Fisher's test P value with outcome as binary phenotype. Additional data file 3 is a figure showing hierarchical clustering over 186 ER^- ^breast cancers (gene annotated version). Additional data file 4 is a figure showing the distribution of basal and ERBB2 markers among ER^- ^subtypes. Additional data file 5 is a table showing the centroids of gene expression for each of the five identified ER- subtypes. Additional data file 6 is a figure showing expression profiles of immune response module genes in ER^- ^samples of the external UPP cohort. Additional data file 7 is a figure showing expression profiles of immune response module genes in ER^- ^samples of the external JRH-2 cohort. Additional data file 8 is a figure showing the clustering of ER+ samples over the humoral immune response gene module in the two external UPP and JRH-2 cohorts.

## Supplementary Material

Additional data file 1 Columns label the gene, the negative kurtosis of its expression profile over 186 ER- samples, the number of clusters predicted by PAC and Fisher's test P value testing for an association between outcome and the two clusters. Click here for file

Additional data file 2 Columns label the gene, the negative kurtosis of its expression profile over 527 ER+ samples, the number of clusters predicted by PAC and Fisher's test P value testing for an association between outcome and the two clusters. Click here for file

Additional data file 3 Hierarchical clustering over 186 ER^- ^breast cancers and 813 negative kurtosis profile genes selected using the PAK algorithm, as explained in the text. Five main clusters were identified and characterized in terms of over-expression of genes related to cell cycle (CC), immune response (IR), extracellular matrix (ECM), and steroid hormone response (SR) functions. Red denotes relative over-expression and green relative under-expression. Click here for file

Additional data file 4 **(A) **Hierarchical clustering dendrogram with the different ER^- ^subtypes as defined by the clustering in Figure 2a. **(B) **The distribution of lymphocytic infiltration scores (LI) and histologic grade. Color codes: black = high LI and high grade; gray = low LI; blue = intermediate grade; and sky blue = low grade. (**C) **Expression profiles of validated basal markers from [27] across ER^- ^subtypes. **(D) **Expression profiles of genes in the *ERBB2 *amplicon. Color codes: green = relative under-expression; red = relative over-expression. Click here for file

Additional data file 5 Table gives the gene expression centroids over the five identified ER- subclasses. Centroids were defined over the 813 genes with negative kurtosis expression profiles. Click here for file

Additional data file 6 Expression profiles (on a log2 scale) of immune response module genes in the validation ER^- ^cohort UPP. Black indicates good outcome samples and red poor outcome samples. Clusters were inferred using the pam algorithm. Inferred clusters are indicated by different shapes (triangles and diamonds). Click here for file

Additional data file 7 Expression profiles (on a log2 scale) of immune response module genes in the validation ER^- ^cohort UPP. Black indicates good outcome samples and red poor outcome samples. Clusters were inferred using the pam algorithm. Inferred clusters are indicated by different shapes (triangles and diamonds). Click here for file

Additional data file 8 Heatmap of gene expression of the 11-gene humoral IR module in the ER^+ ^samples of the **(A) **UPP and **(B) **JRH-2 cohorts. Shown are the clusters over-expressing (purple) and underexpressing (yellow) the humoral IR module as predicted by the pam algorithm. Good outcome samples are presented in gray and poor outcome samples in black. Green indicates relative under-expression, and red relative over-expression. **(C) **Kaplan-Meier survival curves over combined external cohorts (for UPP the end-point was disease-specific survival, and for JRH-2 it was recurrence-free survival), with the number of events and samples in each of the two predicted groups. Click here for file
