# Supplementary figures and images for: An immune response gene expression module identifies a good prognosis subtype in estrogen receptor negative breast cancer
Source: Genome Biol. 2007 Aug 2;8(8):R157. doi: 10.1186/gb-2007-8-8-r157 (PMC2374988; doi:10.1186/gb-2007-8-8-r157)

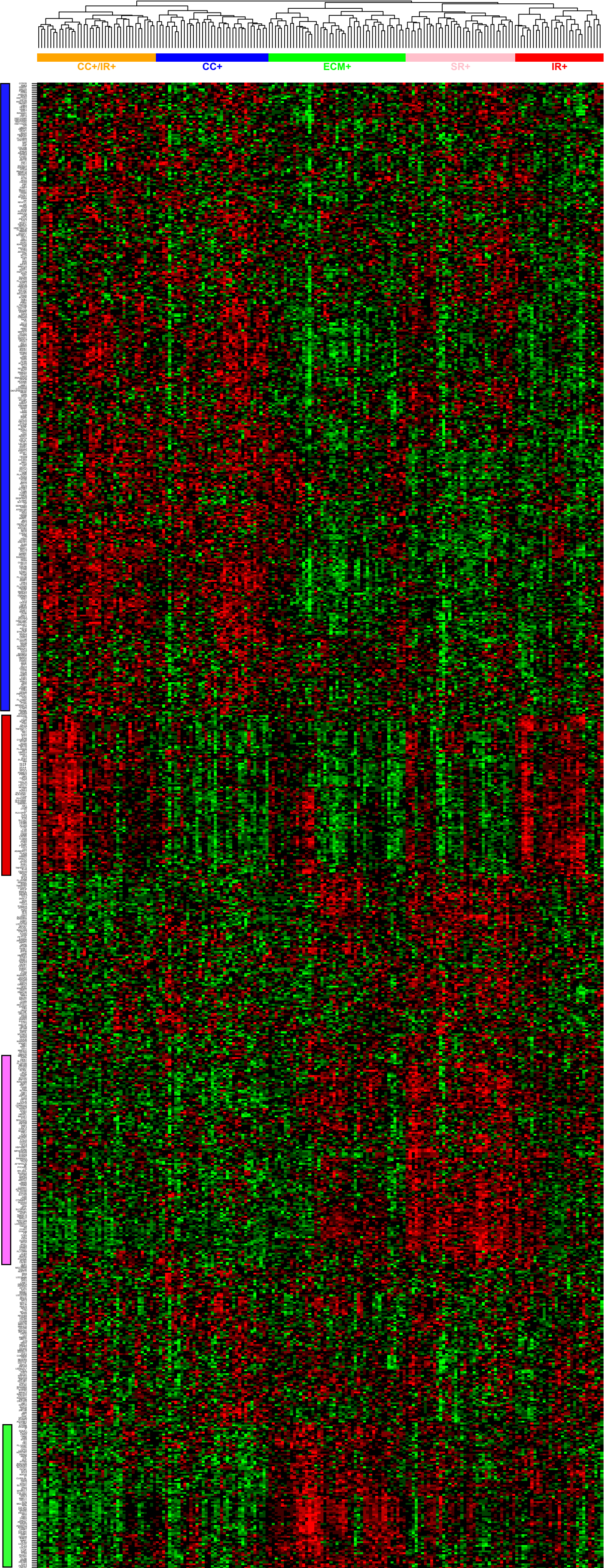

Supplement: Additional data file 3 — Hierarchical clustering over 186 ER- breast cancers and 813 negative kurtosis profile genes selected using the PAK algorithm, as explained in the text. Five main clusters were identified and characterized in terms of over-expression of genes related to cell cycle (CC), immune response (IR), extracellular matrix (ECM), and steroid hormone response (SR) functions. Red denotes relative over-expression and green relative under-expression. [file gb-2007-8-8-r157-S3.pdf]

**A)**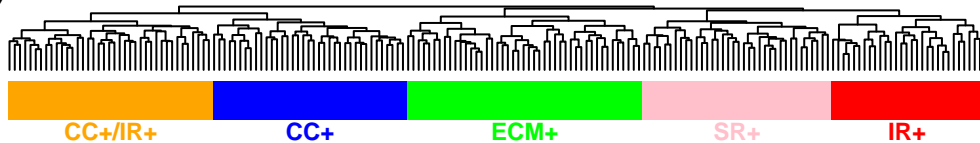**B)**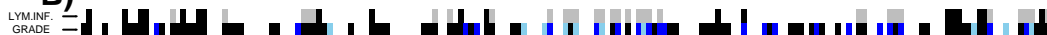**C)**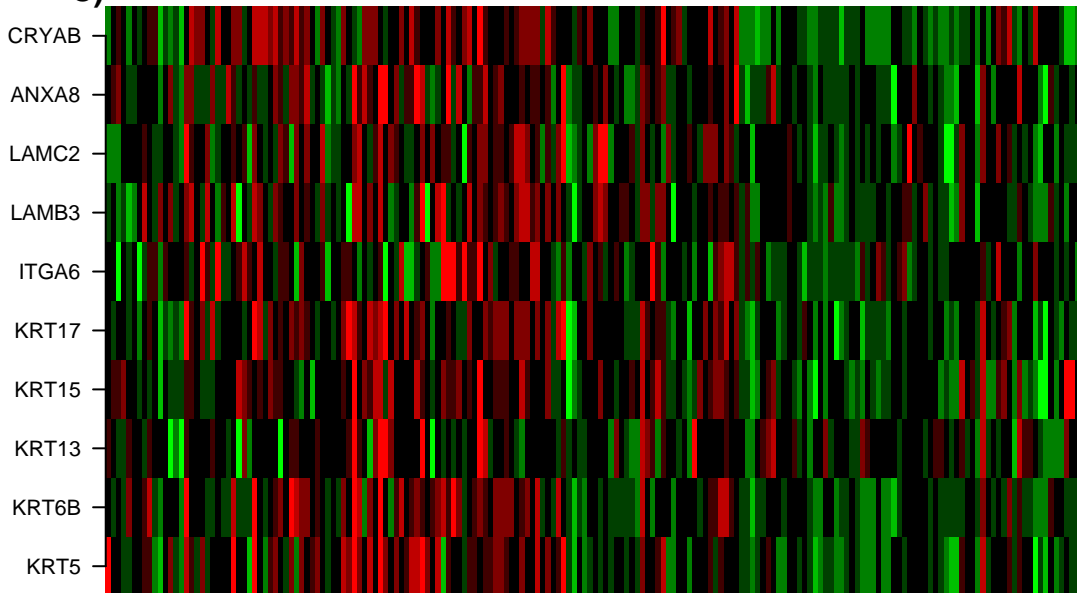**D)**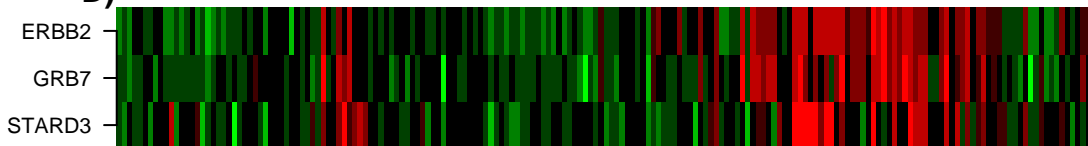

Supplement: Additional data file 4 — (A) Hierarchical clustering dendrogram with the different ER- subtypes as defined by the clustering in Figure 2a. (B) The distribution of lymphocytic infiltration scores (LI) and histologic grade. Color codes: black = high LI and high grade; gray = low LI; blue = intermediate grade; and sky blue = low grade. (C) Expression profiles of validated basal markers from [27] across ER- subtypes. (D) Expression profiles of genes in the ERBB2 amplicon. Color codes: green = relative under-expression; red = relative over-expression. [file gb-2007-8-8-r157-S4.pdf]

**HLA-F-MillerA**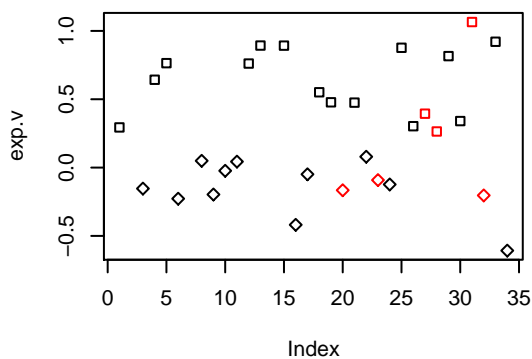**IGLC2-MillerA**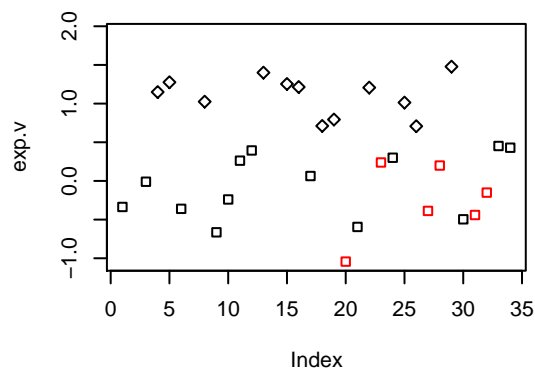**LY9-MillerA**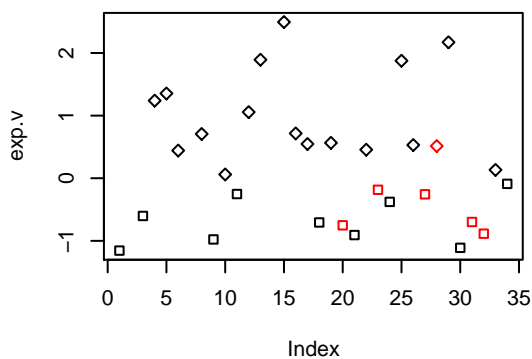**TNFRSF17-MillerA**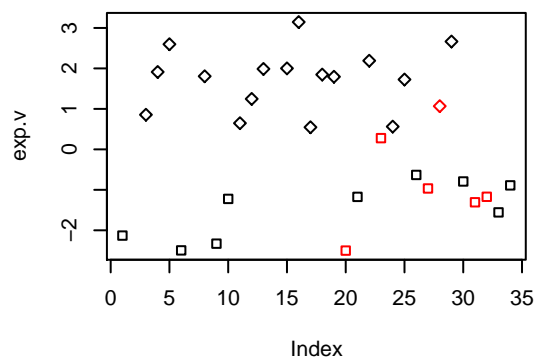**SPP1-MillerA**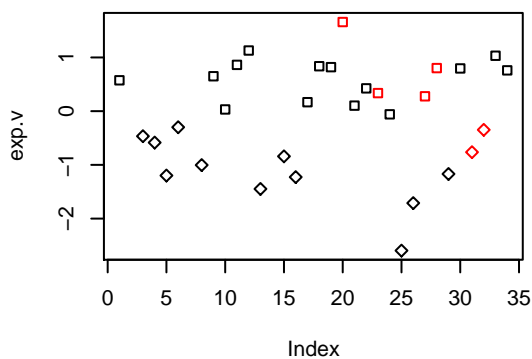**XCL2-MillerA**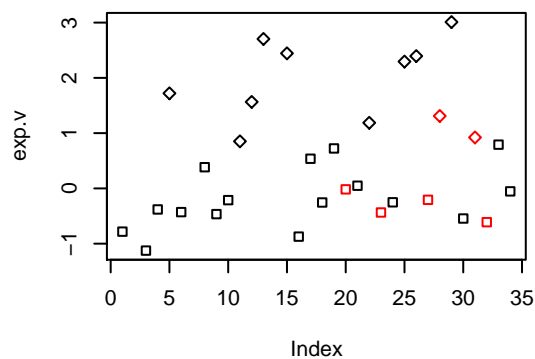**C1QA-MillerA**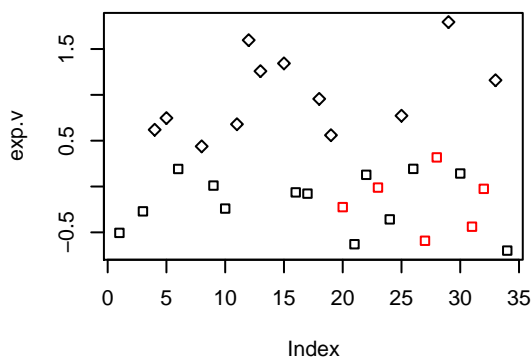

Supplement: Additional data file 6 — Expression profiles (on a log2 scale) of immune response module genes in the validation ER- cohort UPP. Black indicates good outcome samples and red poor outcome samples. Clusters were inferred using the pam algorithm. Inferred clusters are indicated by different shapes (triangles and diamonds). [file gb-2007-8-8-r157-S6.pdf]

**A)**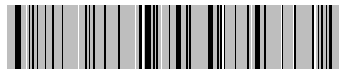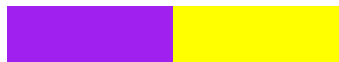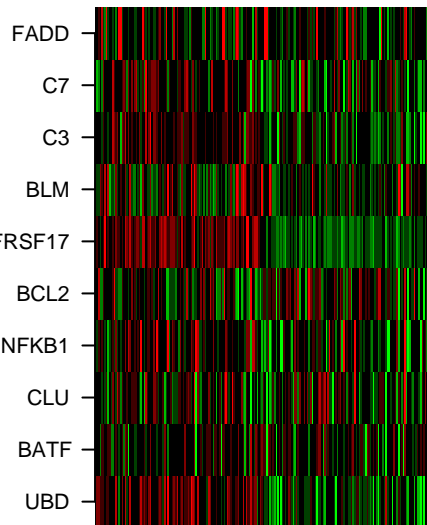**B)**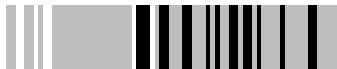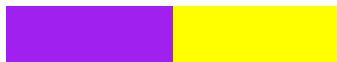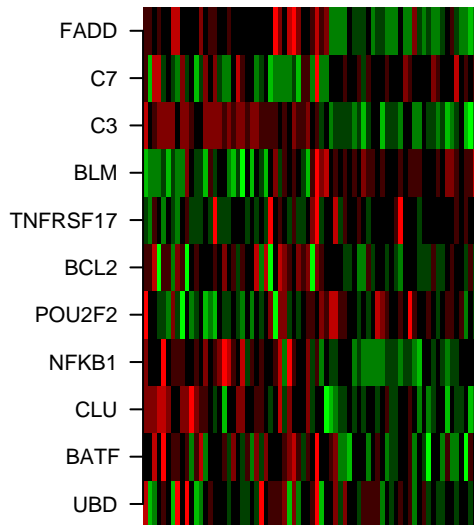**C)**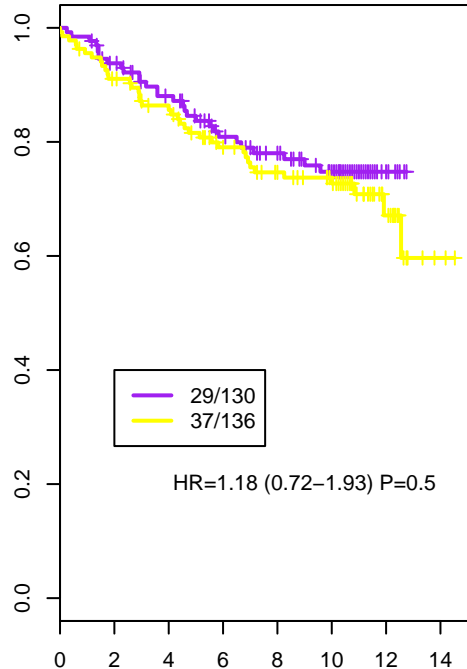

Supplement: Additional data file 8 — Heatmap of gene expression of the 11-gene humoral IR module in the ER+ samples of the (A) UPP and (B) JRH-2 cohorts. Shown are the clusters over-expressing (purple) and underexpressing (yellow) the humoral IR module as predicted by the pam algorithm. Good outcome samples are presented in gray and poor outcome samples in black. Green indicates relative under-expression, and red relative over-expression. (C) Kaplan-Meier survival curves over combined external cohorts (for UPP the end-point was disease-specific survival, and for JRH-2 it was recurrence-free survival), with the number of events and samples in each of the two predicted groups. [file gb-2007-8-8-r157-S8.pdf]
