# Supplementary material for: An immune response gene expression module identifies a good prognosis subtype in estrogen receptor negative breast cancer
Source: Genome Biol. 2007 Aug 2;8(8):R157. doi: 10.1186/gb-2007-8-8-r157 (PMC2374988; doi:10.1186/gb-2007-8-8-r157)
Supplement: Additional data file 7 — Expression profiles (on a log2 scale) of immune response module genes in the validation ER- cohort UPP. Black indicates good outcome samples and red poor outcome samples. Clusters were inferred using the pam algorithm. Inferred clusters are indicated by different shapes (triangles and diamonds). [file gb-2007-8-8-r157-S7.pdf]

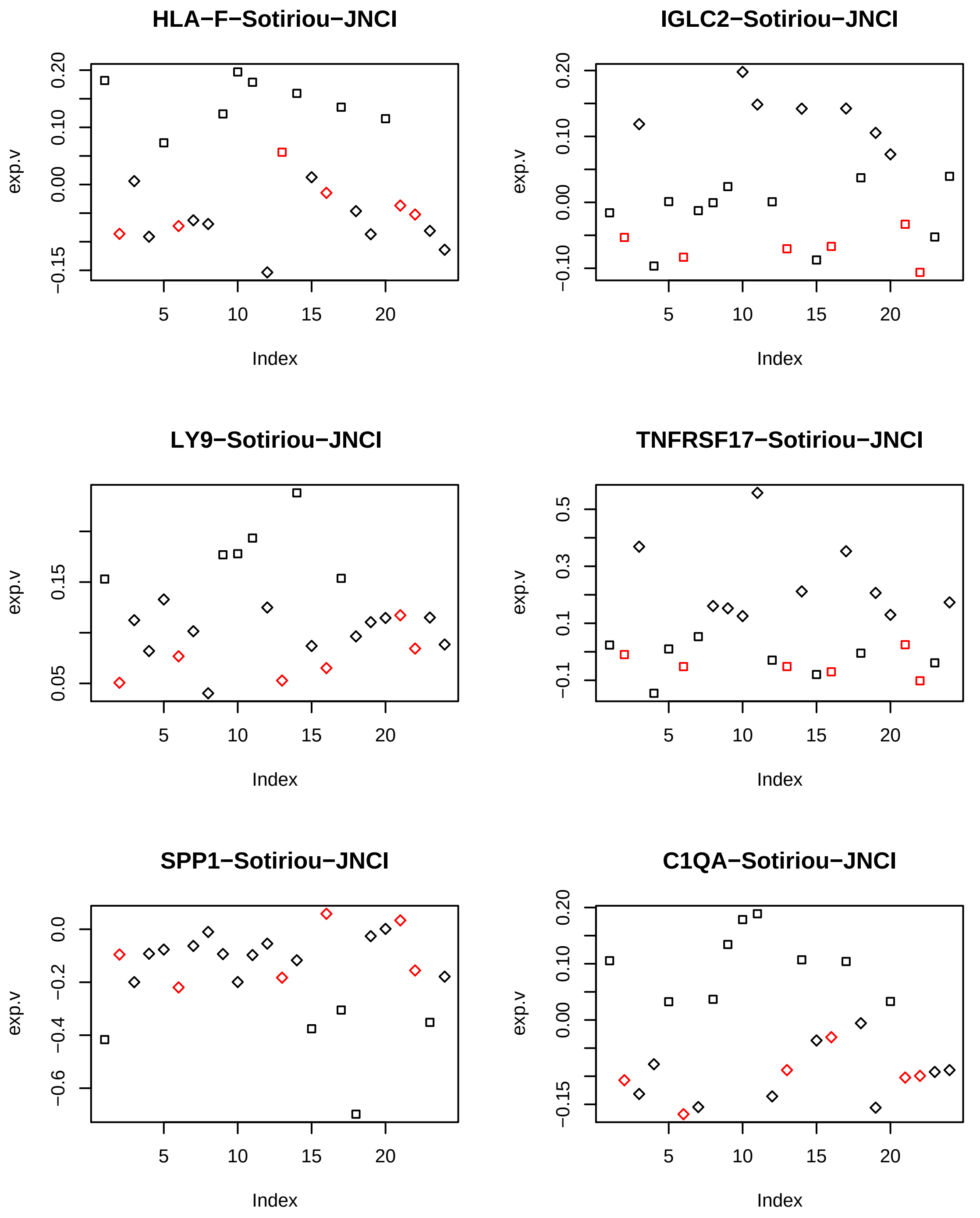

HLA−F−Sotiriou−JNCI
exp.v
−0.15
0.00
0.10
0.20
5
10
15
20
Index
IGLC2−Sotiriou−JNCI
exp.v
−0.10
0.00
0.10
0.20
5
10
15
20
Index
LY9−Sotiriou−JNCI
exp.v
0.05
0.15
5
10
15
20
Index
TNFRSF17−Sotiriou−JNCI
exp.v
−0.1
0.1
0.3
0.5
5
10
15
20
Index
SPP1−Sotiriou−JNCI
exp.v
−0.6
−0.4
−0.2
0.0
5
10
15
20
Index
C1QA−Sotiriou−JNCI
exp.v
−0.15
0.00
0.10
0.20
5
10
15
20
Index
